# Neurophysiological Assessment of Abnormalities of the Neuromuscular Junction in Children

**DOI:** 10.3390/ijms19020624

**Published:** 2018-02-22

**Authors:** Matthew Pitt

**Affiliations:** Department of Clinical Neurophysiology, Great Ormond Street Hospital for Children National Health Service (NHS) Foundation Trust, London WC1N 3JH, UK; matthew.pitt@gosh.nhs.uk

**Keywords:** repetitive nerve stimulation, single fibre electromyography, Myasthenia, Paediatrics, neuromuscular junction

## Abstract

The function of the neuromuscular junction in children is amenable to electrophysiological testing. Of the two tests available, repetitive nerve stimulation is uncomfortable and has a reduced sensitivity compared with single-fibre methodology. The latter is the method of choice, recording the variability in neuromuscular transmission as a value called jitter. It can be performed by voluntary activation of the muscle being examined, which is not suitable in children, or by stimulation techniques. A modification of these techniques, called Stimulated Potential Analysis with Concentric needle Electrodes (SPACE), is well tolerated and can be performed while the child is awake. It has a high sensitivity (84%) for the diagnosis of neuromuscular transmission disorders, the majority of which are myasthenic syndromes, and a moderate specificity (70%). The latter can be improved by the exclusion of neurogenic causes and the determination of the degree of jitter abnormality. Minor jitter abnormalities, under 115% of the upper limit of normal, are usually caused by myopathies with an associated neuromuscular transmission disorder, whereas levels higher than this value are usually associated with one of the myasthenic conditions.

## 1. Introduction

The neuromuscular junction (NMJ) is the site of abnormality in myasthenia. In children, this includes not only autoimmune conditions such as anti-acetylcholine (Anti-ACh) receptor myasthenia and anti-Muscle specific kinase (MuSK) myasthenia, but also congenital myasthenic syndromes (CMS). NMJ function is also disturbed by botulinum toxin, with infantile botulism the most common manifestation in pediatrics [1,2], and by drugs, particularly the neuromuscular blocking agents (NMBA) used in anesthesia and for the paralysis of ventilated patients in the intensive care [3]. The abnormalities of the NMJ cause a disorder in the mechanism by which an action potential passing down the peripheral nerve triggers a chemical reaction in the synapse, producing depolarization of the postsynaptic membrane and a continuation of the action potential down the muscle. Within the presynaptic region, acetylcholine (ACh) is generated by choline acetyltransferase (CHAT) with storage of ACh in vesicles that fuse with the presynaptic membrane to release ACh into the synapse in response to depolarization and the ingress of calcium. Each vesicle contains between 5000 and 10,000 molecules of ACh. ACh binds to the receptors on the postsynaptic membrane causing depolarization, before being broken down by cholinesterase into acetic acid and choline, which is recycled into the presynaptic region. While the autoimmune conditions and drugs affect mainly the postsynaptic regions, the CMS can affect any part of the NMJ. Botulism blocks the release of ACh from the presynaptic region.

Disorders of the NMJ involve changes in membrane potentials and therefore are amenable to neurophysiological techniques. While the knowledge of the genetic abnormalities that cause CMS is increasing exponentially, with new mutations identified regularly, genetic screening for all mutations associated with CMS is not universally available [4]. Another problem with CMS is that their clinical manifestations are protean, unlike the situation in adult myasthenia, and CMS will enter into the differential diagnosis of many different clinical presentations. Amongst these are dysphagia, stridor, sudden infant death, episodic apnea, diaphragmatic weakness, arthrogryposis, and the floppy baby [5,6,7,8]. Without neurophysiological testing, myasthenia may be overlooked as their cause.

In the circumstance where genetic testing for all known mutations is readily available, there may be a reduced role for neurophysiological techniques demonstrating NMJ abnormalities. These techniques may not be needed if the clinical manifestations are typical for one of the known myasthenic conditions and are confirmed by genetic testing. However, in all centers studying myasthenia there is a significant proportion of children, who have clinical and neurophysiological support for a myasthenic condition, but in whom related genetic mutations have not yet been identified. Having confirmation of a NMJ abnormality is important in these cases, as it will encourage clinicians in their pursuit of mutations currently unidentified. Additionally, neurophysiological techniques may be needed to confirm that a particular mutation is causing a NMJ disturbance clinically, if genetic testing has been performed before neurophysiology

## 2. Neurophysiological Techniques

There are two techniques: repetitive nerve stimulation (RNS) and single-fibre (SF) methodology [9]. The latter is divided into voluntarily activated SF electromyography (vSFEMG) and SF techniques where stimulation of the neuromuscular junction is used (StimSFEMG).

### 2.1. Repetitive Nerve Stimulation (RNS)

**Figure 1:**
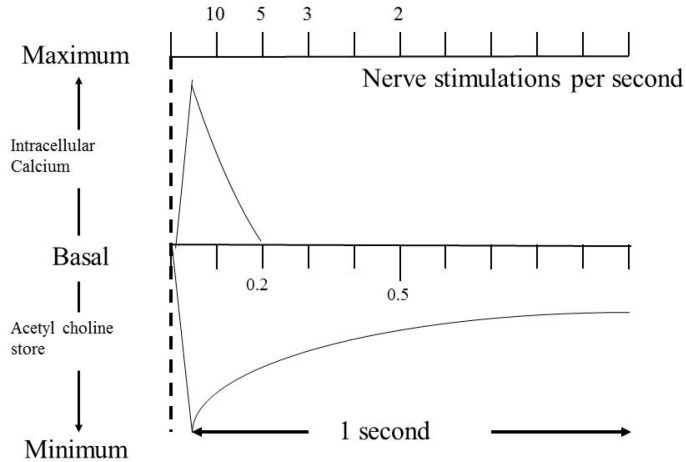
Curves for the recovery of intracellular calcium and acetylcholine stores to their resting state following depolarization of the nerve terminal.

The use of this technique predates SFEMG and has many enthusiasts. There are, however, significant problems with the technique when used in children, firstly it is uncomfortable, and secondly it is positive only when there is a significant degree of neuromuscular transmission abnormality. RNS is administered initially at 3 Hz. This rate is chosen as the response will not be affected by an increased level of calcium in the presynaptic region. Increased calcium intake into the neuromuscular junction will enhance release of ACh. Calcium concentration is restored to the baseline in approximately 0.2 s, therefore rates below 10 nerve stimulations per second will not be complicated by this effect. In contrast, the stores of ACh in the vesicles have a longer half-life and will take longer to be restored to their baseline levels; therefore, provided a rate below 5 nerve stimulations per second is used, the changes seen on RNS at 3 Hz are only those from the depletion of ACh quanta (Figure 1). 

When the neuromuscular junction is stimulated at 3 Hz, the number of quanta of ACh released follows a set pattern, with the maximal amount being released after the first stimulation and decreasing over the next three to four stimulations, when it reaches a nadir before returning to a higher level, which is lower than that after the first stimulation. End-plate potentials (EPPs) mirror exactly the number of quanta released. Because the EPPs exceed the threshold for depolarization of the postsynaptic membrane by a significant degree in healthy synapses, the so-called “safety factor”, this decrement of the EPPs seen around stimulation number 5 is not noticeable in the resulting muscle action potential (MAP), which, once the threshold is exceeded, is an all-or-nothing response. The sensitivity of repetitive nerve stimulation is enhanced by stimulating the neuromuscular junction tetanically, that is over 10 Hz, which will cause an enhanced increase of calcium uptake, which in turn will cause increased release of ACh. This is the cause of the so-called post-tetanic facilitation. Again, in healthy subjects, since the “safety factor” is so high, this will show no change in the resultant MAP. The post-tetanic facilitation is followed by the phenomenon of post-tetanic exhaustion when the amount of ACh available to be released has been depleted because of the enhanced calcium input, and this will cause the EPPs to drop. Again, in healthy subjects this has no effect on the MAP.

**Figure 2:**
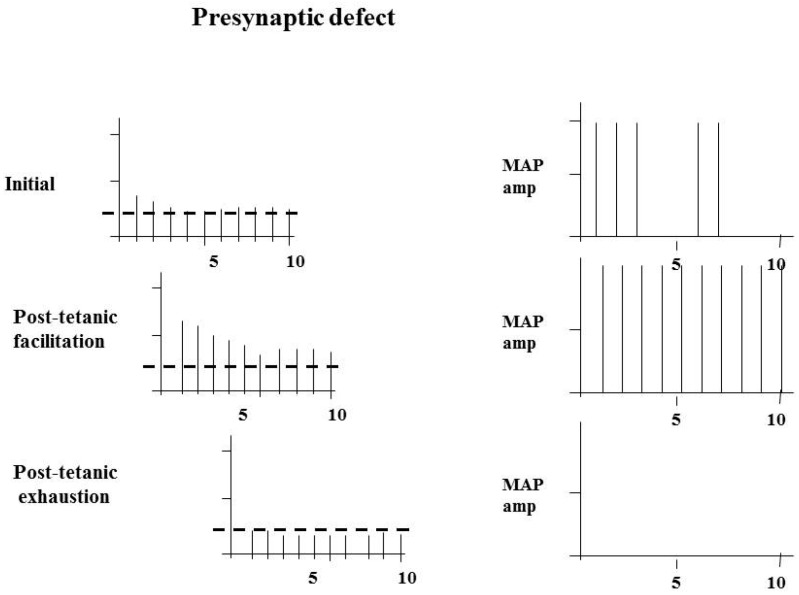
The effects of a presynaptic abnormality of the neuromuscular junction (NMJ) with reduced numbers of acetylcholine (ACh) quanta released and, consequently, reduced end-plate potentials. The threshold for depolarization is indicated by the dashed line. The initial study may show a failure of muscle fibre depolarization at the fourth or fifth stimuli. A significant improvement occurs with post-tetanic facilitation, with deterioration in the period of post-tetanic exhaustion. MAP: motor action potential.

The RNS abnormalities in synapse disorders are best understood when considering the effects of pre- and postsynaptic abnormalities separately. Both disorders affect the “safety factor”, but by different means. In a presynaptic abnormality the amount of ACh released is reduced, while the threshold remains the same. The baseline studies at 3 Hz may show a failure of depolarization at the fourth or fifth stimulus, or they may be normal. Post-tetanic facilitation may correct any abnormality that is present by increasing the total amount of ACh released, but in the period after this, with post-tetanic exhaustion, the abnormality will become much more pronounced, with the “safety factor” reduced significantly throughout most of the stimulations. Therefore, in a presynaptic abnormality, the baseline studies may be normal or abnormal. Tetanic stimulation, by increasing the amount of ACh released in those motor endplates where the safety factor is reduced, will improve the situation, and each stimulation will be followed by a MAP. The compound muscle action potential (CMAP), which is the summation of all the motor action potentials, will show a marked increase with tetanic facilitation, commonly to over 100% of the first response. In the post-tetanic exhaustion phase, the abnormality will become more pronounced, and RNS will show a clear abnormality (Figure 2).

With a postsynaptic abnormality, the threshold is increased in the postsynaptic region but the sequence of changes is similar to those seen in the presynaptic abnormality, that is, at baseline with stimulation at 3 Hz there may be no abnormality, or, if an abnormality is present, there will be a reversal of the abnormality after post-tetanic facilitation and an augmentation of the abnormality in the post-exhaustion stage. The difference between the pre- and postsynaptic abnormalities is that the compound muscle action potential will be low in the former and show the marked enhancement described, which is not seen in the postsynaptic abnormality. The important neurophysiological feature in these studies is that the RNS abnormality is only present if there is a failure of transmission of the nerve action potential arriving at the presynaptic region, across the synapse, to the postsynaptic membrane.

These changes described are those in individual muscle fibres, but, clearly, the CMAP will reflect the summation of the changes seen in all of the muscle fibres in the muscle in question. Some will be normal and some will be markedly affected, but the abnormality will reflect the changes described, namely, with a decrement first appearing at the potential 4 or 5 in the train of stimulations. Normality of the RNS is considered if the fourth or fifth potential is within 10% of the baseline.

The technique in children is relatively easy to perform, although uncomfortable. It is important to choose a weak muscle, and proximal muscles are more likely to be affected than distal muscles. The trapezius is relatively easy to study, whereas the anconeus is usually difficult. One of the major difficulties in children is movement. As a rule, if, during repeated studies, a reproducible abnormality appears to be present but one study is normal, this usually means that the RNS is normal and the remainder of the abnormalities artifactual.

### 2.2. Single-Fibre EMG (SFEMG)

**Figure 3:**
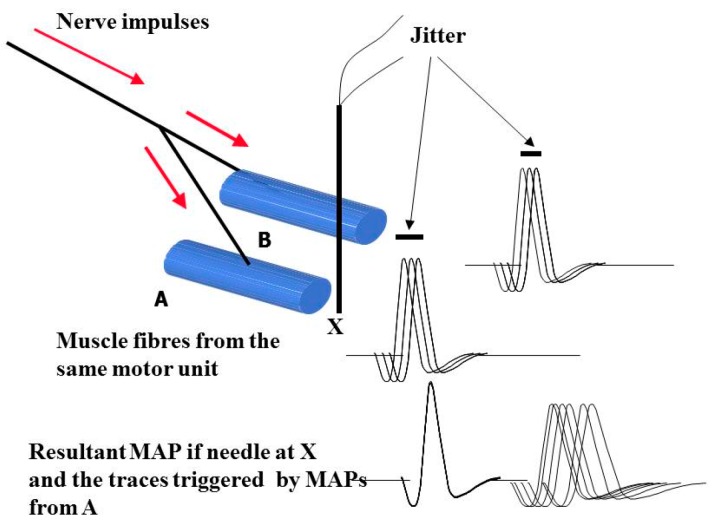
Single-fibre electromyography (EMG). MAPs from two musclefibres from the same motor unit showing the variability of the MAP latency with jitter due to repeated stimulation. When the recording screen is triggered by the MAP from fibre A, the jitter is the summation of two muscle fibres’ jitter.

When performing EMG with a conventional needle, MAPs from many different muscle fibres are recorded. Up to 10 muscles fibres are usually within the range of the needle, but these may come from different motor units. SFEMG allows those MAPs from fibres that come from the same motor unit to be isolated by either reducing the surface area of the recording needle, which is the mechanism used with the SFEMG needle, or altering the low-frequency filter to exclude MAPs from distant fibres. In the technique of vSFEMG the subject is asked to contract the muscle gently and the needle is positioned to record from muscle fibres from the same motor unit. These fibres will fire together as the nerve impulses pass down the nerve branches to the different muscle fibres, but there will be a slight variation with repeated stimulation. This slight variation is called jitter. This is shown in the next figure (Figure 3). Voluntary SFEMG is nearly impossible in a child under eight years of age, even when highly experienced practitioners do it. It certainly cannot be consistently performed in children that young and therefore does not form a basis for a screening technique. StimSFEMG removes the need for voluntary contraction and gives a greater opportunity of success in children, and is the preferred technique.

#### The Problems with Nomenclature in Single-Fibre EMG

**Figure 4:**
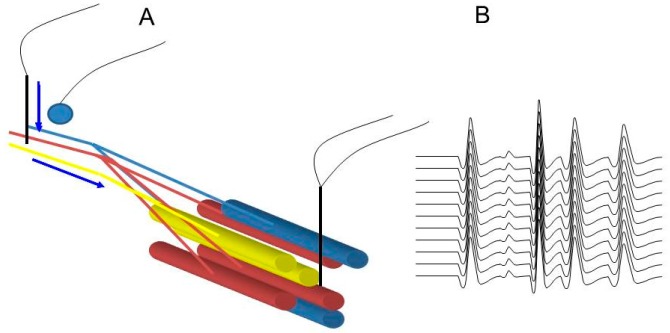
Principles of stimulated single-fibre EMG. Motor fibres from several different motor units are stimulated by a monopolar needle in reference to a surface recording (**A**). The action potentials recorded from the concentric needle electrode placed in the muscle originating from different muscle fibres of several motor units (**B**).

In principle, stimulating a nerve and recording from the muscle underpins the technique of StimSFEMG. Unfortunately, because it is impossible to limit the number of muscle fibres that may be stimulated by this technique, it is very uncommon to record MAPs that originate from single muscle fibres. Instead, because of the overlap of the MAPs, the operator will be recording a small action potential rather than a single fibre’s potential (Figure 4). While there are descriptions of the techniques that might ensure selection of those potentials coming from single muscle fibres, these are only valuable if the study is normal [10]. As soon as abnormalities of the NMJ become more severe, the possibility of overlap of MAPs from muscle fibres from different motor units increases. If strict criteria defining a single-fibre potential are used, no measurement can be made despite the fact that the test is manifestly abnormal. For this reason, our group has moved away from calling the technique that we use StimSFEMG, instead naming it SPACE, an acronym for Stimulated Potential Analysis with Concentric needle Electrodes [11,12].

The choice of needle has been forced on the neurophysiological community by worries about prion and other related conditions, which prevent the use of reusable needles. SFEMG needles are very expensive, and therefore few departments can afford to use them as single-use items. Consequently, the use of concentric needle electrodes with an enhancement of the low-frequency potential filter has been the method of choice in the last few years. Using a small-diameter concentric needle electrode, the so-called facial needle, and a high low-frequency filter, an approximation of the technique of SFEMG has been made. If there were concerns that with a conventional SF needle the potentials were not single-fibres’, this situation is not improved by the use of the facial needle. This is another reason why we prefer the term SPACE.

## 3. The Use of SPACE in Children

The muscle chosen for examination with this technique is the orbicularis oculi. This is for two reasons; firstly, it is commonly affected by nearly all of the known causes of NMJ dysfunction in children, although some of the more recently diagnosed CMS caused by disorders of glycosylation, such as those caused by mutations in the *GMPPB* gene, are characterized by limb-girdle localization and may not have abnormalities in orbicularis oculi [13]. The second reason is that this muscle is approached from behind the head, in a way similar to the method used by dentists, which means that the child will be unaware of what is being done. A local anesthetic cream is used to damp down the sensation, although the child will still feel the stimulation and also the pressure, as the needle is passed into the muscle.

**Figure 5:**
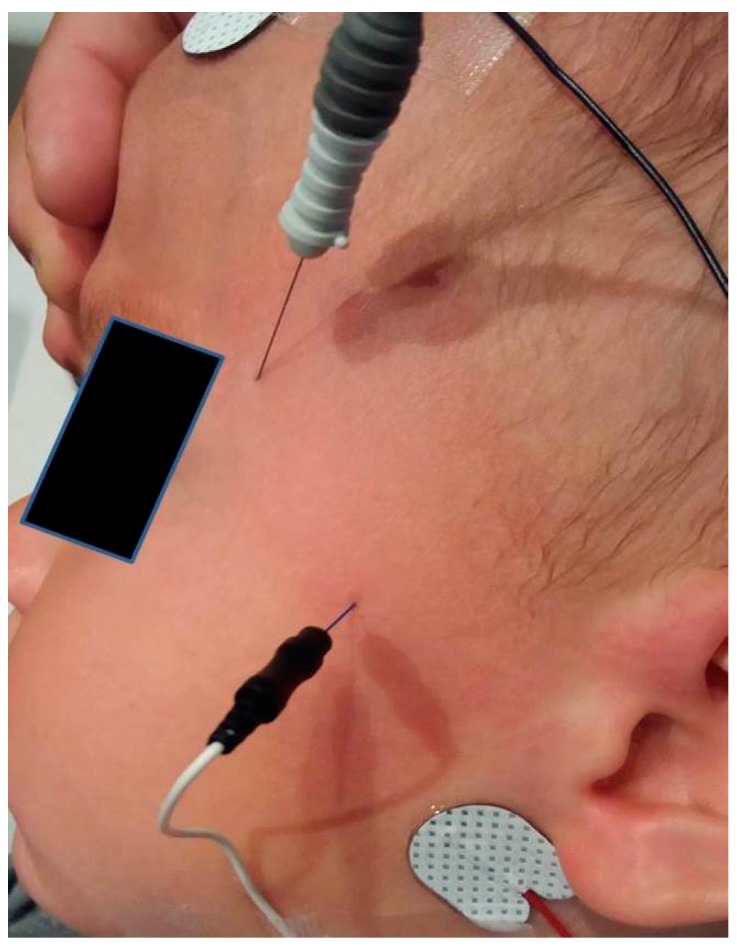
Position of the stimulating and recording electrodes when assessing jitter in the left orbicularis oculi.

A monopolar needle of very fine diameter (0.35 mm 28 French Gauge) is used to stimulate the facial nerve as it passes over the zygomatic arch. This is referenced to a surface electrode to produce bipolar stimulation. An earth electrode is placed on the forehead, and a concentric needle electrode is placed at the outer canthus to record the fibres from orbicularis oculi. This is shown in Figure 5.

To increase the chances of success, it is important to see a clear activation of orbicularis oculi before inserting the recording electrode. If this cannot be seen, it is important to either reposition the needle or, if still unsuccessful, remove it and place it once more in the skin. The rule is to keep the stimulation potential below 1 mA to be sure that the stimulating needle is close to the nerve. In this circumstance, the response in the muscle is an all-or-nothing response with very small changes in the stimulus, producing a stable muscle potential. After an initial stimulation with single shocks, trains of stimuli are given at 10 Hz, and the potentials are recorded accordingly. While it is recommended that one should see a hundred stimulations of each potential, this is rarely achieved in adults and never in children. Normally, around 20 stimuli suffice. The needle is then moved slightly, and another population of muscle fibre potentials is obtained. The aim is to collect a minimum of 20 potentials.

## 4. Normative Data

Ethics committees are unlikely to allow this test to be performed on entirely normal children as it is invasive and, even with a local anesthetic, uncomfortable. Attempts to obtain normative data from anesthetized children may encounter resistance if a perception that the anesthetic is in any way altered to obtain the data is sustained. It is therefore fortunate that a technique which allows normative data to be drawn down from routine attendees at the laboratory exists. This is called extrapolated norm (e-norms) methodology and is detailed in several papers by Prof Joe Jabre in collaboration with the author of the present work [14,15]. The technique is well described in these papers, and the reader is directed to them. Using this technique, we found that the neuromuscular junction appears to be very mature at an early age and that, after two years of age, the upper limit of normal stabilizes at 26 µs. Under that age, even if we have encountered children as young as six weeks of age, who have changes that are indistinguishable from a teenager, most children do have a slightly less mature neuromuscular junction, and the upper limit of normal is 45 µs under one year of age, and 32 µs under two years but over one year of age.

## 5. Results of Examination with SPACE

Our experience over a period since 2007 is detailed in two review articles [11,12]. The conclusions of these studies are that the technique has a sensitivity of 84% for the detection of abnormalities in conditions known to cause NMJ dysfunction, which include patients with myasthenia but also others with botulism and persistence of NMBA effects (Table 1). It has a moderate specificity of 70%. The sensitivity is relatively low considering the whole group, but this is influenced by a proportion of those cases being due to some forms of myasthenia such as GMPPB, known not to affect the orbicularis oculi [13]. Sensitivity values when looking at autoimmune myasthenia and particular CMS such as that associated with Rapsyn mutation, are very high, exceeding 90%. Specificity has always been a problem with stimulated techniques of single-fibre assessment, because any abnormality between the point of stimulation at the nerve and the muscle may cause an abnormality.

Neurogenic disorders of the muscle are easy to recognize by other techniques used in EMG, and if these common causes of jitter abnormality are removed, the specificity improves marginally to 74%.

A certain proportion of muscle disorders have been shown to have abnormalities of neuromuscular transmission [16,17,18,19,20]. Fortunately, most of these tend to be associated with minor disorders of jitter, while most of the myasthenic conditions in children, including the paralysis by botulinum toxin, have significant abnormalities of jitter. A receiver operator curve assessment of our data showed that, if the measurement is greater than 115% of the upper limit of normal for age, it is highly likely that the underlying condition is myasthenia, provided a neurogenic abnormality has been excluded; if the measurement is below that level but above normal, it is more likely that the condition is a myopathy with an associated neuromuscular transmission disorder (NMTD). The discrimination of the findings by the jitter value allows the clinician to direct further investigations accordingly. If the abnormality of the jitter is above 115% of normal, an assessment for myasthenia is the first line of examination, while, in the reverse situation, a muscle biopsy may be more productive.

## 6. Conclusions

While repetitive nerve stimulation has enthusiastic support in adults, it is too uncomfortable to be easily applied to children, and in any case, both theoretically and empirically, has reduced sensitivity for diagnosing neuromuscular junction disorders, as demonstrated when the results are compared in a cohort of children with proven myasthenic conditions (see Table 2).

The preferred technique, which we describe under the acronym SPACE, is a modification of StimSFEMG. In our hands, it has very high acceptability for both parents and children alike and can be performed on children without the need for a general anesthetic. It has high sensitivity and good specificity, the latter increased by removing those cases with a neurogenic abnormality and not considering as myasthenia those cases with minor jitter abnormalities. In this situation, a myopathic process with a neuromuscular transmission disorder is more likely to be the underlying cause.

## Figures and Tables

**Table 1:** Results of SPACE in primary disorders of the NMJ.

Diagnosis	Number	Number (%) with Abnormal MCD
AIMG (Anti-MuSK *n* = 2)	23	21 (91)
Congenital Myasthenic Syndromes (CMS)	46	39 (85)
Dok 7	15	15 (100)
ColQ	9	8 (89)
CHRNE	4	4 (100)
Rapsyn	3	3 (100)
CHRNG	3	2 (67)
Slow channel syndrome	2	2 (100)
GFPT1	2	2 (100)
GMPPB	7	2 (29)
Agrin	1	1 (100)
Probable Myasthenia (not otherwise specified)	25	19 (76)
Neuromuscular Blocking Agents (NMBA)	3	3 (100)
Botulism	9	7 (78)
Primary NMJ Diagnoses (Total)	106	89 (84)

Legend: MCD = mean consecutive difference (synonymous with jitter) Dok7 = Docking protein 7. ColQ = collagen like tail subunit of asymmetric acetylcholinesterase. CHRNE = ACh receptor epsilon subunit mutation. CHRNG = ACh receptor gamma subunit mutation. GFPT1 = glutaimine-fructose-6-phosphate transaminase 1. GMPPB = GDP-mannose pyrophosphorylase B.

**Table 2:** Comparison of the results of repetitive nerve stimulation and SPACE in definite myasthenia, proven either genetically or by autoantibodies.

		Repetitive Nerve Stimulation		
		Positive	Negative	
SPACE	Positive	29	18	47
	Negative	1	0	1
Total		30	18	48
